# Veterans health administration national cardiac device surveillance program: Structured, centralized remote monitoring for cardiovascular implantable electronic devices to improve quality of care

**DOI:** 10.1016/j.hroo.2025.03.024

**Published:** 2025-04-08

**Authors:** Sanket S. Dhruva, Kurt Marunick, Hans J. Moore, Gregory Rohrbach, Thomas L. Rotering, Merritt H. Raitt

**Affiliations:** 1 Section of Cardiology, Department of Medicine, San Francisco Veterans Affairs Health Care System, San Francisco, California, USA; 2 Cardiology Section, Medical Service, Washington, DC Veterans Affairs Medical Center, Washington, DC, USA; 3 Veterans Affairs Portland Health Care System, Portland, Oregon, USA

**Keywords:** Cardiovascular implantable electronic device, Implantable cardioverter-defibrillator, Implantable loop recorder, Pacemaker, Patient adherence, Remote monitoring

## Abstract

Remote monitoring (RM) is central to care of patients with cardiovascular implantable electronic devices (CIEDs). However, because RM care presents a large and increasing clinician workload, solutions are needed to increase care efficiency. The Veterans Health Administration National Cardiac Device Surveillance Program (VHANCDSP) is a response to this need by centralizing RM processes, reviewing all RM transmissions, identifying patients affected by recalls, and providing guidance on clinical response for 122 VHA CIED clinics across the United States and Puerto Rico. The VHANCDSP promotes participation and adherence in RM, ensuring evidence-based care. The centralized infrastructure also positions the VHANCDSP to evaluate and implement quality improvement interventions, ultimately enhancing clinical care for patients with CIEDs. This article describes and reflects on this infrastructure and provides specific recommendations about how high-quality RM care can be provided in care other health care systems.

**Key Points**

▪ Managing remote monitoring care of patients with cardiovascular implantable electronic devices (CIEDs) represents a large clinical workload.
▪ Centralized remote monitoring through the Veterans Health Administration National Cardiac Device Surveillance Program (VHANCDSP) improves evidence-based remote monitoring care.
▪ VHANCDSP promotes remote monitoring adherence while reducing unnecessary transmissions.
▪ VHANCDSP ensures highly accurate, timely, and cost-effective evaluation of remote transmissions.
▪ These learnings can inform other health systems about optimizing CIED care.

## Introduction

Pacemakers and implantable cardioverter-defibrillators (ICDs) are recommended by clinical practice guidelines for various indications.^1^, ^2^, ^3^, ^4^, ^5^, ^6^ These devices monitor and treat potentially lethal cardiac arrhythmias, improving patient outcomes and quality of life. Together with implantable loop recorders (ILRs),^7^ cardiovascular implantable electronic devices (CIEDs) play an increasingly important role in clinical care, with more than 1.7 million implanted annually worldwide.^8^

Follow-up care for patients with CIEDs centers on remote monitoring (RM), the process of transmitting data acquired and stored by the CIED in response to a schedule, preset alert criteria, or patient initiation. RM is recommended as standard-of-care as a Class 1, Level of Evidence A recommendation because randomized clinical trials (RCTs) and large observational studies have demonstrated improved clinical outcomes through early detection of actionable findings such as arrhythmias, lead failure, and battery depletion.^8^ RM also reduces the need for in-person evaluations and is cost effective.^8^ The widespread adoption of RM into clinical practice^9^ was accelerated during the COVID-19 pandemic, as its value in enhancing patient access to care was recognized.^10^^,^^11^ Increasing adoption of RM, however, has added substantial administrative and clinical workload for front-line clinicians to handle the vast amount of incoming data while also creating opportunity to leverage associated datasets for quality improvement and research initiatives.^8^

## Challenges with RM and the device clinic

The first step to achieving benefits from RM is to ensure that patients are enrolled in the RM system and, among patients who are enrolled, maintaining connectivity (for patients with CIEDs that have wireless telemetry capability) and adherence to scheduled remote transmissions (for all patients, but in particular those who must manually send transmissions). However, some patients are not enrolled,^12^ and even those who are enrolled may become disconnected or nonadherent to RM.^13^ These issues are magnified when responding to a manufacturer alert or Food and Drug Administration (FDA) recall, which may necessitate urgent CIED assessment. Ensuring consistent RM connectivity is a Class 1 recommendation for alerted or recalled devices to enable early detection of actionable events.^8^

Next, to ensure that patients with CIEDs can achieve the many benefits of RM,^8^ clinicians must review and act upon RM transmissions as clinically appropriate. Transmissions triggered by alert settings are most likely to have clinically significant findings, but they require skilled human interpretation and timely management decisions. Alert volume is very high^14^; in 1 large RM cohort of 26,713 patients with CIEDs, 55% of patients had ≥1 alert transmission over a 1-year period, amounting to 82,797 alerts.^15^ In fact, more than 40% of all RM transmissions were alert-initiated.^15^ To compound challenges, although many RM transmissions are repetitive or redundant, even those without alerts may contain important findings that require clinical action. Therefore, reducing the burden of nonactionable alert transmissions is important, as is ensuring that potentially important findings are not missed.

Highlighting these multiple challenges of RM, a recent survey of allied health professional clinicians found that 47% did not think that their clinic was adequately staffed to handle the large volume of RM reports, and a similar percentage was not satisfied with workflows for responding to alerts, including patient follow-up.^16^ The biggest challenges identified in managing patients with RM were the high number of transmissions, maintaining RM connectivity, and inadequate staffing.^16^

Accordingly, there is a need to structure a system in which the enormous and growing RM workload in caring for a population of patients with CIEDs is effectively managed through support provided to both front-line CIED clinicians who focus on clinical decision making and to patients. As noted in the preceding paragraphs, the key challenges are ensuring patient enrollment, maintaining RM connectivity, reviewing transmissions and ensuring timely appropriate clinical actions, and reducing unnecessary alert burden. Within the Veterans Health Administration (VHA), a large integrated health care delivery system in the United States, the National Cardiac Device Surveillance Program (VHANCDSP) seeks to address these challenges while providing centralized care for more than 68,000 patients. We review VHANCDSP’s processes and offer generalizable recommendations for efficient, successful centralized RM systems that could be implemented by other health systems.

## VHANCDSP patient population and RM transmission volume overview

**Figure 1:**
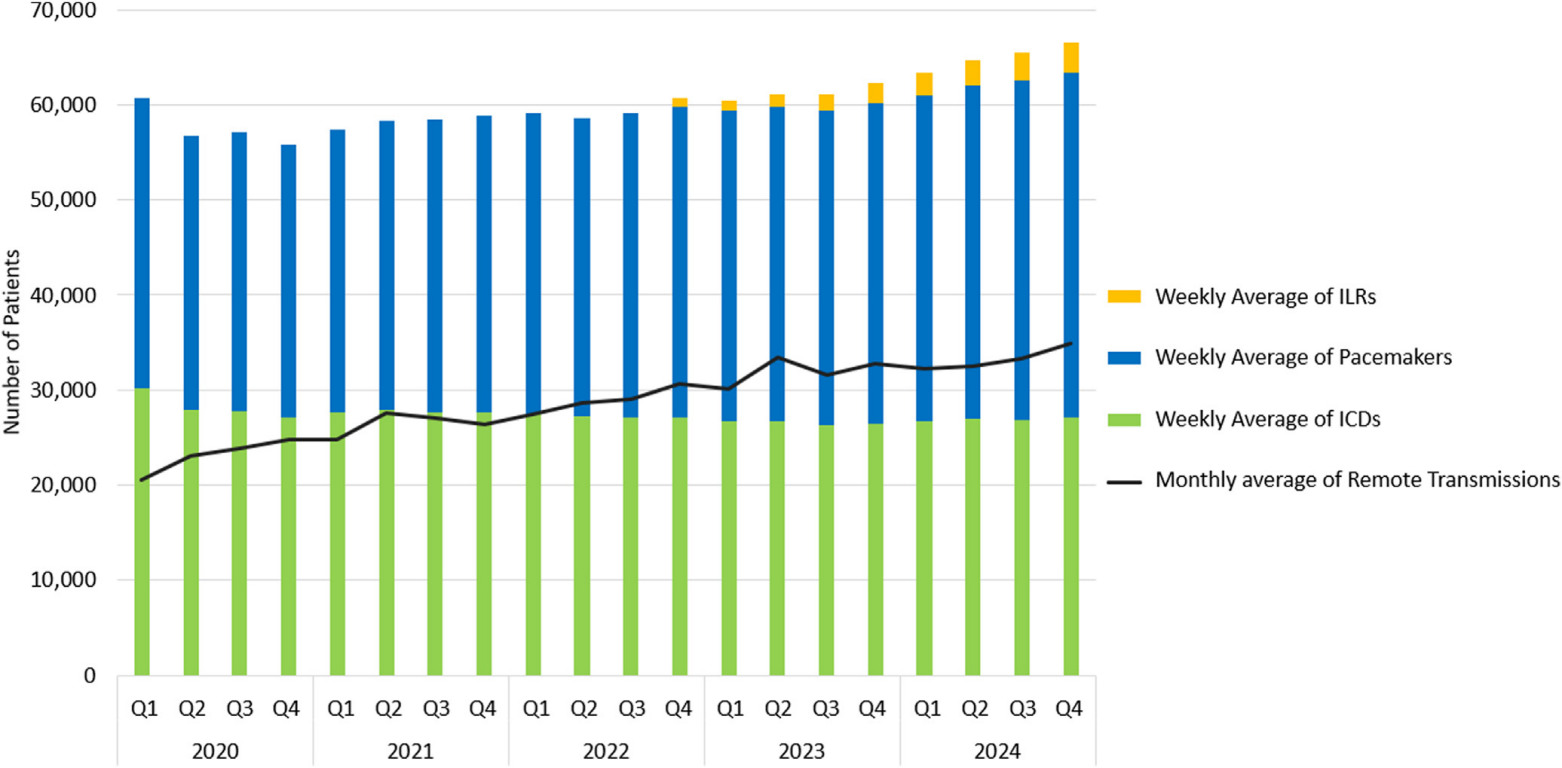
Number of patients enrolled in remote monitoring and remote transmissions received, 2020–2024. ICDs = implantable cardioverter-defibrillators; ILRs = implantable loop recorders.

In 2024, the VHANCDSP reviewed 379,111 remote transmissions for 68,967 patients at 122 clinics (Figure 1). These numbers have increased from 262,203 transmissions from 53,276 patients in 2020. The relative increase in the number of transmissions relative to the patients enrolled has been driven primarily by increased adherence with RM.

Among the overall population of 68,131 patients as of December 2024, the mean (standard deviation) age of the patient population was 75.1 (10.3) years, and 64,569 (94.8%) were male (Table 1). Overall, 51,157 (75.1%) were White and 10,811 (15.9%) were Black or African American; 23,886 (35.0%) resided in rural locations. In terms of comorbidities, 36,333 (53.3%) had heart failure, 37,962 (55.7%) had atrial fibrillation (AF), and 41,034 (60.2%) had coronary artery disease. The most common CIED type was pacemakers in 33,233 (48.9%), followed by ICDs in 16,780 (24.6%), cardiac resynchronization therapy-defibrillators (CRT-Ds) in 10,437 (15.3%), CRT pacemakers in 3506 (5.1%), and ILRs in 4204 (6.2%).

**Table 1:** Characteristics of patients actively engaged in remote monitoring through the Veterans Health Administration National Cardiac Device Surveillance Program

	Total (n = 68,131)
Age, mean ± standard deviation (years)	75.1 ± 10.3
Gender	
Female	2988 (4.4%)
Male	64,569 (94.8%)
Prefer not to answer/transgender woman/transgender man/nonbinary	574 (0.8%)
Race	
American Indian or Alaska Native	442 (0.6%)
Asian	337 (0.5%)
Black or African American	10,811 (15.9%)
More than 1 race	497 (0.7%)
Native Hawaiian or other Pacific Islander	453 (0.7%)
White	51,157 (75.1%)
Prefer not to answer, unknown, or missing	4434 (6.5%)
Ethnicity	
Hispanic or Latino	3322 (4.9%)
Not Hispanic or Latino	61,640 (90.5%)
Prefer not to answer, unknown, or missing	3169 (4.7%)
Rurality	
Urban	44,217 (64.9%)
Rural	23,062 (33.8%)
Highly rural	824 (1.2%)
Prefer not to answer, unknown, or missing	28 (0%)
Geographic region	
Continental	13,791 (20.2%)
Northeast	19,068 (28%)
Pacific	12,230 (18%)
Southeast	23,006 (33.8%)
Outside United States	14 (0%)
Prefer not to answer, unknown, or missing	22 (0%)
Comorbidities	
Heart failure	36,333 (53.3%)
Atrial fibrillation	37,962 (55.7%)
Chronic kidney disease	29,281 (43.0%)
Diabetes mellitus	34,160 (50.1%)
Valvular heart disease	4316 (6.3%)
Chronic obstructive lung disease	18,518 (27.2%)
Coronary artery disease	41,034 (60.2%)
Peripheral vascular disease	10,519 (15.4%)
Stroke or transient ischemic attack	8002 (11.7%)
Device type∗	
Pacemaker	33,323 (48.9%)
Implantable cardioverter-defibrillator	16,780 (24.6%)
Cardiac resynchronization therapy: defibrillator	10,437 (15.3%)
Cardiac resynchronization therapy: pacemaker	3506 (5.1%)
Implantable loop recorder/implantable cardiac monitor	4204 (6.2%)

∗ A total of 119 patients have multiple cardiovascular implantable electronic devices.

## CIED registration and RM participation

### Overview

**Figure 2:**
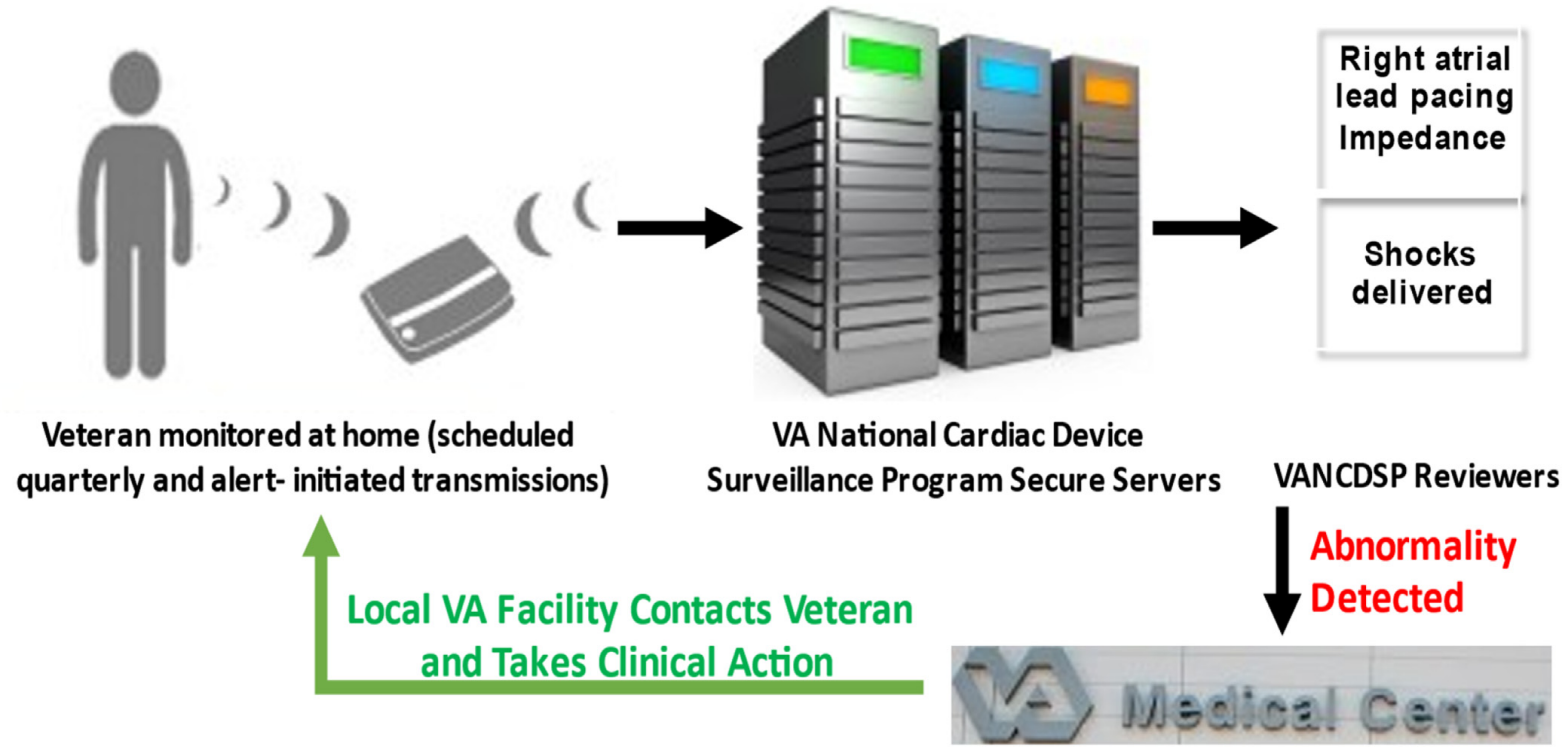
Remote monitoring process within Veterans Health Administration.

The VHA operates 122 CIED clinics across the United States and Puerto Rico, providing comprehensive local CIED patient management. A VHA Directive mandates that all patients who receive ongoing follow-up for their CIEDs by any of these 122 VHA clinics must be registered with the VHANCDSP,^17^ whether their CIED was implanted in a VHA hospital or outside VHA. CIEDs from all major manufacturers are available for implantation in VHA. This registration is performed through a Web-based application and includes generator and lead information (Figure 2), ensuring that patients affected by manufacturer alerts or FDA recalls are identified.^17^ Patient information is updated when there is a CIED or component change to avoid gaps in both RM and population management. Although local clinicians cannot directly make changes on CIED manufacturer websites—to ensure consistency and accuracy in the central database—VHANCDSP processes any requested changes within 1 business day and makes multiple dashboards that aggregate data across patients from all CIED manufacturers, as described here. The VHANCDSP automatically screens all registered devices to determine if they are affected be a recall and—if they are—highlights these devices.

In addition to registration, the VHA Directive mandates that all local VHA clinics ensure that willing and able patients are offered enrollment in RM, and this RM must take place through the VHANCDSP’s centralized RM infrastructure.^17^ This system provides data consistency and reduces errors, improving patient safety and freeing local CIED clinicians to focus on direct patient care.

To ensure that patients who are followed for CIED care in VHA clinics are not inadvertently missed, the VHANCDSP also checks each VHA CIED clinic every 3 months for Current Procedural Terminology (CPT) codes for in-person CIED interrogation. Lists of patients who have been seen and with a CPT code for CIED interrogation, but who are not registered with the VHANCDSP, are provided to local VHA clinics to prompt patient registration, if appropriate.

### Results

Active participation in RM is essential for patients to achieve clinical outcome benefits of RM. VHANCDSP tracks participation, defined as the percentage of patients followed by a given VHA CIED clinic who participate in RM. The VHA target for each of the 122 clinics is ≥80% of followed patients to participate in RM. The current overall participation rate nationally is 87%, with 94 (77.0%) of the clinics having >80% of patients with CIEDs participating and 55 (45.1%) of the clinics having >90% of patients with CIEDs participating.

### Recommendations (Table 2)

Although mandates for centralized RM are not likely in most health systems, the experience of VHANCDSP demonstrates the importance of offering RM to all patients at the time of implant or when patients transfer their CIED care into the practice through the use of standardized operating procedures (SOPs); Expert Consensus recommends RM be initiated before discharge or within 2 weeks of implant.^8^ Further, examining administrative claims data or electronic health records for patients who may have been seen in a CIED clinic and then periodically (eg, quarterly) reviewing these patient lists can help to ensure that all patients are asked to participate in RM.

**Table 2:** Recommendations for optimal remote monitoring care

Area of CIED care	Recommendations
Remote monitoring participation	Mandating that all patients are offered remote monitoring at the time of implant Checking claims or electronic health records to identify patients seen in a clinic to ensure that they are followed in remote monitoring
Remote monitoring adherence and connectivity	Directly reaching out to nonadherent or disconnected patients and asking them to use manufacturer resources to reconnect Providing adherence data to clinicians
Remote transmission review	Periodic reviews to ensure accuracy Standardized operating procedures for notification to local device clinicians
Reducing nonactionable transmissions	Modification of alerts Examining frequently transmitting patients and modifying alerts or requesting patients to curtail sending patient-initiated transmissions Reprogramming implantable loop recorders for false positive transmissions

## RM adherence and connectivity

### Overview of transmission scheduling and receipt

Once patients are enrolled in RM through the VHANCDSP, they are scheduled to send RM transmissions at least once every 90 days. Transmissions may also be scheduled more frequently if there is a specific clinical need, such as surveilling AF burden. For CIEDs without wireless telemetry capability approaching their elective replacement indicator (ERI), RM transmissions are scheduled monthly. Patients who must send manual transmissions, generally those with pacemakers implanted many years ago, receive postcards approximately 7 to 14 days before the date of their scheduled transmissions to notify them to transmit on that date.

Through direct data connections with all 4 major CIED manufacturers, discrete data and PDFs for every remote transmission are automatically transferred to the VHANCDSP database. This means that once a patient sends an RM transmission, the data are immediately available. The 7-terabyte VHANCDSP database goes back to the 1990s, with more than 3.2 million remote transmissions.

### Maintaining RM adherence

RM adherence is defined as the percentage of patients participating in RM who sent transmissions within the past 100 days, a period that allows a 10-day buffer beyond the prescheduled transmissions every 90 days. As recommended by Expert Consensus, VHANCDSP has established processes to ensure that patients maintain adherence and connectivity.^8^

Informed by interviews with patients about RM,^18^ and supported by the VHA Measurement Science Quality Enhancement Research Initiative (QUERI), VHANCDSP also has developed, studied, and now employs multiple strategies to improve patient adherence. These strategies come in 2 forms: direct-to-patient outreach and providing data resources for front-line VHA clinicians. The direct-to-patient outreach includes postcards that are mailed to patients who have previously transmitted but have not sent transmissions in 110 days (allowing an additional 10-day buffer after the 100 days for adherence). These postcards notify patients that a transmission has not been received, the date of the patient’s last RM transmission, and brief information about the clinical benefits of RM. The postcards advise patients to call the manufacturer of their CIED for assistance and provide the manufacturer-specific toll-free phone number. For patients with pacemakers or ICDs from manufacturers other than Biotronik (Berlin, Germany), patients are asked to then send a manual transmission so that they can be placed on a regular schedule. In an RCT of 6351 patients followed by the VHANCDSP, these postcards, along with 1 additional postcard approximately 1 month later for patients who still did not transmit, were found to more than double the rate of adherence at 10 weeks.^19^ In contrast, a separate RCT, also conducted by the VHA Measurement Science QUERI and VHANCDSP, found that mailing postcards or letters to patients with pacemakers or ICDs who had not sent a transmission from those devices for ≥1 year, and in most cases had never sent an RM transmission, lacked success^20^; thus, postcards are only sent to patients who have recently transmitted. Given that nearly all patients now have wireless CIEDs in which maintaining connectivity is the primary goal, the VHANCDSP has recently studied mailing postcards to patients who have become disconnected from RM for ≥16 days. Overall, these direct-to-patient efforts support patients in improving RM adherence by leveraging manufacturer resources and— importantly—without burdening local VHA CIED clinicians.

**Figure 3:**
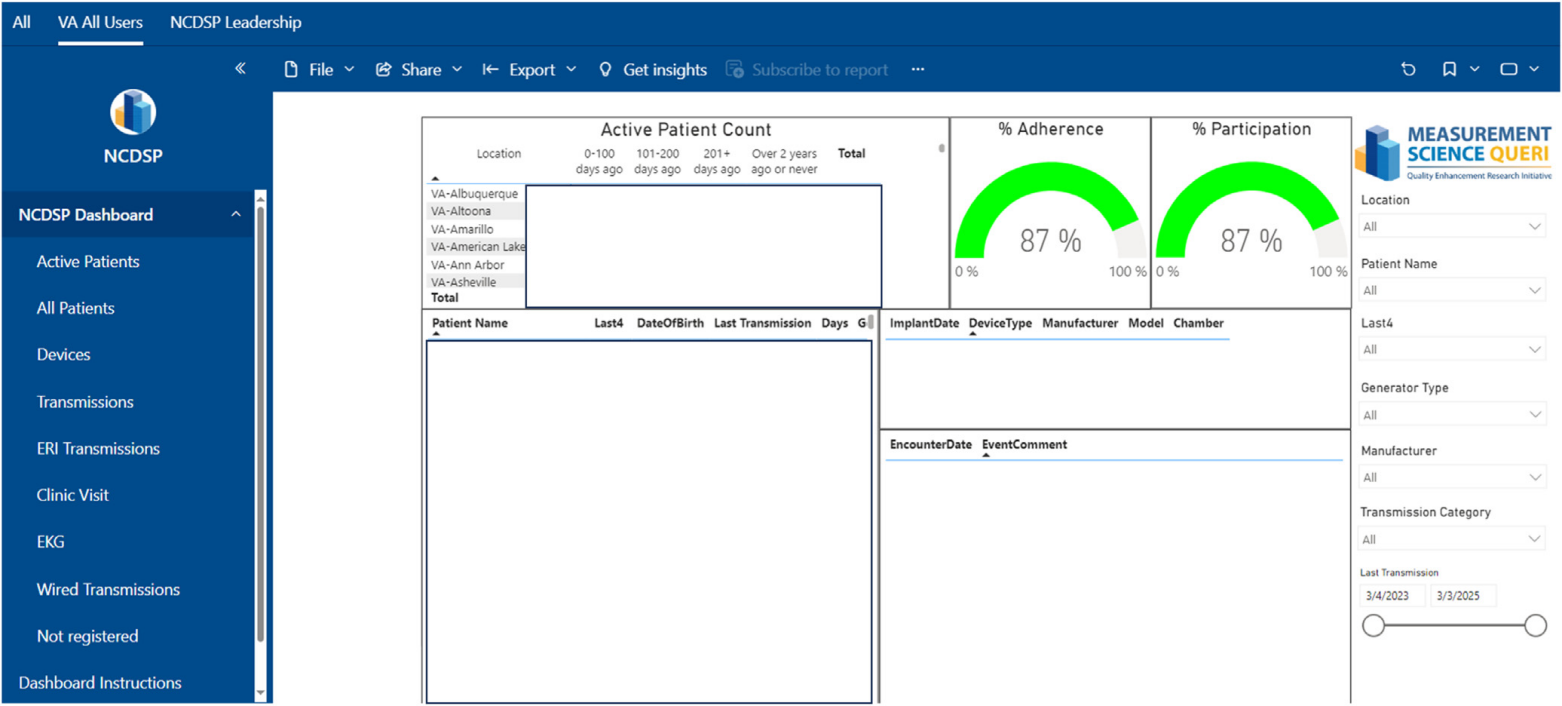
Veterans Health Administration National Cardiac Device Surveillance Program dashboard, developed by Veterans Health Administration Measurement Science Quality Enhancement Research Initiative.

**Figure 4:**
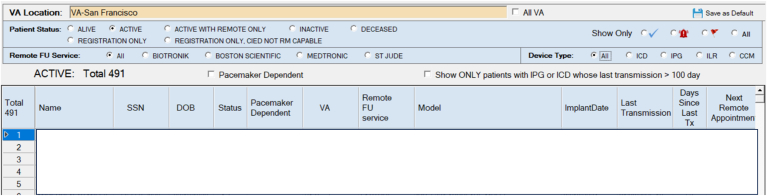
Veterans Health Administration National Cardiac Device Surveillance Program application.

VHANCSDP also supports front-line VHA clinicians in identifying patients with low adherence in a standardized manner. Multiple dashboards (Figure 3 and Figure 4), including one developed by the VHA Measurement Science QUERI, are made readily available to inform local VHA clinicians when a patient last sent an RM transmission, obviating the need to manually check individual CIED manufacturer websites. Given the focus on maintaining consistent and continuous RM connectivity, VHANCDSP now obtains daily connectivity data from all CIED manufacturers and plans to provide these on a VHA dashboard. Finally, also supported by the VHA Measurement Science QUERI, the VHANCDSP has surveyed and interviewed 27 front-line clinicians to identify best practices for RM adherence^21^ and disseminated these findings of emphasizing patient education, regularly assessing and addressing nonadherence, using staff protocols, and engaging CIED manufacturers. Feedback was targeted to CIED clinics with suboptimal adherence.

To emphasize the importance of high participation and adherence, both these quality metrics are made available at quarterly intervals to VHA leadership, in addition to being available on multiple dashboards.

### Results

**Figure 5:**
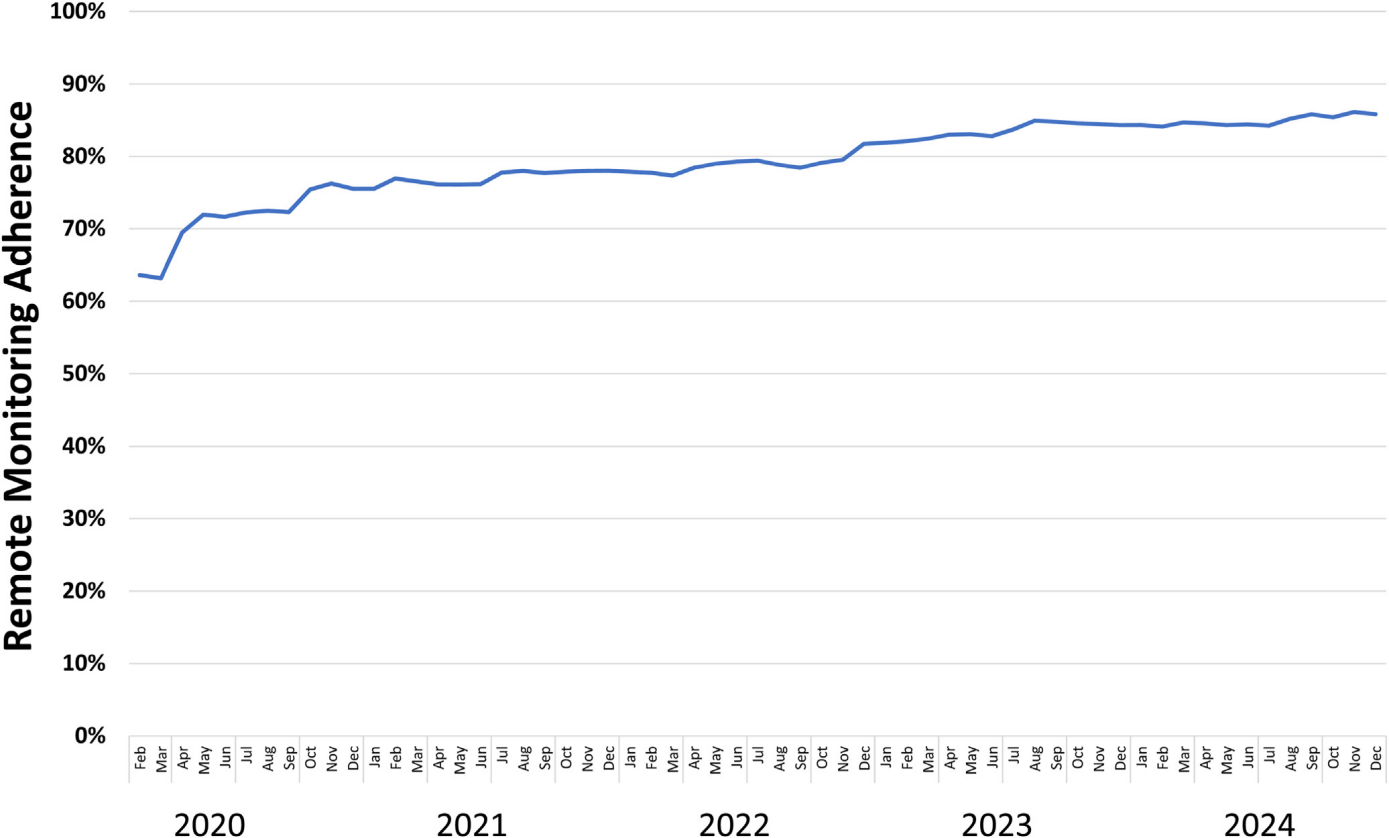
Change in remote monitoring adherence per month, February 2020 to December 2024. Note: Remote monitoring adherence is defined as a patient sending a transmission at least once within the past 100 days.

The VHA target for each of the 122 clinics is ≥80% of patients will send a transmission within the preceding 100 days. The current overall adherence rate nationally is 87%, with 99 (81.1%) of clinics having >80% adherence and 31 (25.4%) of clinics having >90% adherence. VHANCDSP’s efforts have been associated with significant increases in RM adherence over time (Figure 5). These results compare favorably with adherence in settings outside of VHA, although definitions of adherence are variable.^22^ That said, there are still reasons for nonadherence; interviews conducted with veterans followed by VHA, most of whom were not fully adherent, identified gaps including a need to discuss RM and its clinical outcome benefits as well as how to send a transmission, and patients losing their monitors.^18^ Further, it is likely that other factors, such as prolonged hospitalizations or patients traveling without their transmitters, also limit adherence with RM. Finally, ensuring adherence also requires tremendous staff time, which can be difficult to find for clinicians who are already very busy with multiple competing responsibilities.

### Recommendations (Table 2)

The experience of VHANCDSP demonstrates the importance of both direct-to-patient outreach and using CIED manufacturer resources to assist patients in ensuring RM adherence. Given that manufacturers have the most expertise in their RM systems and the substantial time investment that ensuring adherence can take for CIED clinicians,^16^ this strategy is particularly important. At the same time, clinicians need access to information to support adherence, such as checking during routine CIED clinic visits to ensure that patients are transmitting. Future initiatives should also examine electronic communication to ensure RM adherence and connectivity, which is especially important, given the increasing use of smartphone-based RM.^23^

## RM transmission review

### Overview of centralized review process and communication with local clinics

To ensure timely review of RM transmissions, the VHANCDSP employs 20 nurses and CIED technicians whose sole responsibility is to review all RM transmissions each business day. Alert transmissions are prioritized and reviewed within 1 business day, consistent with professional society consensus recommendations,^8^ and nonalert transmissions are reviewed within 3 business days. Based upon VHANCDSP internal quality-assessment review of 4658 scheduled no events transmissions from CIEDs manufactured by Medtronic (Minneapolis, MN), it was determined that these normal transmissions do not require review, although many are still reviewed as workload permits.

If VHANCDSP nurse or CIED technician reviewers identify RM findings expected to be clinically actionable, they follow a standard operating procedure, which stipulates that notification be sent to the local VHA clinician workgroup responsible for following the patient, along with documentation of the relevant RM transmission (Table 3). Documentation of RM transmissions is also made available through the VHA’s electronic health record and through a Web-based VHANCDSP application. Local VHA clinicians are then responsible for taking clinical action, as appropriate, which is generally each business day.

**Table 3:** Findings for which local Veterans Health Administration clinics may receive notification

AFL/AF episodes noted
AFL/AF noted, longest episode 30 seconds to <6 minutes
AFL/AF noted, longest episode 6 minutes to <24 hours AFL/AF noted, longest episode ≥24 hours
AFL/AF noted, persistent (>7 days)
Treated AT/AF episode(s)
False AT/AF detection
VT/VF on the presenting electrogram
VT detected in the monitor zone
Inappropriate VT/VF detection in a monitor zone
VT/VF inappropriately called SVT by discriminators
VT/VF in therapy zone terminated prior to therapy
VT/VF with ATP
VT/VF with shock(s)
VT/VF with ATP and shock(s)
Inappropriate shock therapy (+/– ATP)
Inappropriate ATP therapy
VT/VF induced by ATP or shock
Suspected bradycardia pacing induced VT/VF
Appropriate discrimination of SVT
High V rate episodes
High V rate episodes, non-sustained VT
High V rate episodes, SVT
High V rate episodes, AFL/AF with rapid ventricular response
High V rate episodes, oversensing
BiV pacing <90%
Cumulative Right Ventricular Pacing > 20%
Battery at ERI
Battery at EOL
RV lead impedance out of range
LV lead impedance out of range
Atrial lead impedance out of range
HV/SVC lead impedance out of range
Possible RV failure to capture
Possible LV failure to capture
Possible atrial lead failure to capture
Possible lead failure
Ventricular undersensing
Atrial undersensing
Ventricular oversensing
Atrial oversensing
Possible generator failure
High pacing threshold
Oversensing: suspect make–break potentials
Oversensing: suspect myopotentials in bipolar sensing
Oversensing: suspect myopotentials in unipolar sensing
PVC burden >10%
Appropriate brady episode(s)∗
Appropriate pause(s)∗
Inappropriate brady episode(s)∗
Inappropriate pause(s)∗

AF = atrial fibrillation; AFL = atrial flutter; ATP = antitachycardia pacing; BiV = biventricular; ERI = elective replacement indicator; EOL = end of life; HV = high-voltage; LV = left ventricular; PVC = premature ventricular complex; RV = right ventricular; SVC = superior vena cava; SVT = supraventricular tachycardia; VF = ventricular fibrillation; VT = ventricular tachycardia.

∗ Implantable loop recorders only.

Further, through its Web-based application (Figure 4), the VHANCDSP helps local CIED clinicians with review of remote transmissions. PDFs and discrete data elements from all 4 major CIED manufacturers are made accessible and sortable by manufacturer, date, and alert urgency. Clinicians can view tables and graphs of battery and lead parameters and examine arrhythmia episodes. The application also facilitates the creation of local clinician notes to supplement VHANCDSP review of remote transmissions, allowing clinicians to track which transmissions they have reviewed without separately going to each manufacturer’s website.

### Ensuring accurate review of RM transmissions

Accurate adjudication of RM transmissions is essential, particularly considering the screening role of VHANCDSP readers. To maintain high standards, all readers participate in a quarterly review of 25 RM transmissions, each containing at least 1 abnormality. These abnormalities are randomly selected from key RM categories such as lead-related issues, AF, and ICD therapy. This review process is supervised by a cardiac electrophysiologist. Should readers' accuracy fall below the specified threshold, they receive additional training and monthly reviews continue until their accuracy meets the benchmark.

### Cost effectiveness

Based on current levels of RM adherence in the VHANCDSP patient population and anticipated Medicare-level reimbursement for only the technical codes of pacemakers, ICDs, and ILRs review, the VHANCDSP would enable approximately 25% additional revenue compared with expense for personnel.

### Recommendations (Table 2)

Maintaining the highest level of accuracy is essential to ensure that local clinics can rely on RM transmission review. VHANCDSP’s experience is that SOPs for notifications ensures that local clinicians know the findings for which they can expect to receive notification, while also ensuring that the remote transmission data are easily available for review to inform decision-making.

## Reducing nonactionable transmissions

### Nonactionable alert transmissions

Although participation and adherence in RM are critical, research has shown that repetitive alert and patient-initiated transmissions can compound workload.^24^ In an effort to minimize nonactionable alert transmissions, readers follow a defined set of VHANCDSP guidelines that aligns with professional society consensus recommendations^8^ to make iterative adjustments to RM alert settings, thereby reducing workload on VHANCDSP staff and the local clinics.

VHANCDSP readers do not provide notification to clinics when alerts are ongoing. For example, if a patient develops AF >24 hours, the VHANCDSP will notify the local VHA clinic up to twice. At that point, the manufacturer alert can be turned off, and clinics do not need to be notified again for AF >24 hours. However, if local VHA clinics request notification of another episode of AF (such as if the patient has pursued a rhythm-control strategy with antiarrhythmic medications or ablation), the clinic can email the VHANCDSP to provide repeat notification, and the VHANCDSP will do so.

### High transmission volume and patient-initiated transmissions

Each month, the VHANCDSP identifies patients who have transmitted >7 times in the past 30 days to determine if these transmissions are alert-initiated or patient-initiated. Approximately one-half are alert-initiated, and one-half are patient initiated. If alert-initiated, alerts are adjusted as appropriate. If patient-initiated, their local CIED clinicians are notified and asked to contact the patient to provide education and address individual concerns.

### ILRs

ILR monitoring has traditionally been challenging, given the high volume of false positive transmissions and need for in-person visits for reprogramming.^15^^,^^25^ Over the past 3 years, with the development and availability of remote reprogramming for ILRs paired with algorithms to reduce false positives, the VHANCDSP evaluated the utility of these ILR features in clinical practice and found that monitoring could be successfully and safely performed. In the evaluation, remote interrogations were scheduled every 90, instead of 30, days to decrease overall transmission burden because alerts should detect any clinically pertinent findings.^26^ VHANCDSP now offers local VHA clinics to have their patients with remotely reprogrammable ILRs monitored through the VHANCDSP. Although VHANCDSP monitoring of ILRs is not mandatory, 92 (75.4%) of 122 VHA CIED clinics currently have their patients with remotely reprogrammable ILRs monitored by the VHANCDSP.

### Recommendations (Table 2)

Reducing nonactionable transmissions, both alert-initiated and patient-initiated, is critical to effective workflow. A centralized RM system must take active steps to reduce nonactionable transmissions, both through updating alert settings and contacting patients when needed.

## Optimizing overall CIED care

### Ensuring optimal care delivery by local clinicians

In addition to participation and adherence data, through linkage to VHA’s national electronic health record, VHANCDSP's dashboard (Figure 3) makes available information about in-person cardiology and CIED clinic visits to ensure that patients are not lost to follow-up. Local VHA clinics determine the frequency of in-person evaluations; most perform in-person evaluations every 12 months, and some clinics more frequently (eg, every 6 months). Given that generator replacement at ERI is essential, VHANCDSP also makes available information about patients who have a generator at ERI but who have not yet had a new generator placed. Through providing these data, VHANCDSP seeks to ensure that any gaps in patient care are promptly identified, reducing the risk of missed or delayed care.

### CIED safety

By having all patients with CIEDs followed by VHA clinics registered, the VHANCDSP plays an important role in safety monitoring because patients with recalled CIEDs can be quickly identified and notified and recall-specific actions can be initiated. When such safety issues arise, the VHANCDSP sends each clinic a list of all patients followed by their clinic who are affected and marks patients with affected devices on the Web-based application that is accessible to the local VHA clinicians. The VHANCDSP sends recommendations to address the recall and provides a method to track when specific actions must be taken, such as reprogramming patients’ therapy zones. VHANCDSP works with VHA’s National Center for Patient Safety integrating FDA, manufacturer, and expert guidance into recall management. VHANCDSP has also begun tracking remediation efforts, such as the proportion of patients whose ICDs and CRT-Ds were reprogrammed and the time to reprogramming according to manufacturer recommendations after the 2022 and 2023 Medtronic Class 1 FDA recalls.^27^ The Web-based application allows clinicians to see which patients have or have not had their programming updated.

### Recommendations

Although centralized RM has many benefits and is a central part of the management of patients with CIEDs, clinicians must also be supported to ensure that other aspects of CIED care are optimized. Given that timely decision-making to address CIED component recalls is essential to maintaining patient safety, centralized RM systems also provide an opportunity to ensure a coordinated response to implementing recommendations.

## Generating evidence to improve care

The large size of the number of patients with CIEDs also enables evidence generation that can inform and improve clinical practice. For example, previous research using the VHANCDSP database identified a higher failure rate among the Riata and Riata ST (St Jude Medical, Minneapolis, MN) ICD leads and informed a Class 1 (highest severity) FDA recall.^28^ VHANCDSP data were also used in a collaborative study with FDA that demonstrated how RM data could be used to strengthen assessment of lead failure, particularly for those leads that do not undergo revision or replacement.^29^

Similarly, implementation and effectiveness trials are enabled by leveraging the centralized VHANCDSP infrastructure. VHANCDSP partnered with 2 VHA CIED clinics to implement use of a CIED-based heart failure prediction tool in routine clinical practice, providing the foundation for an RCT.^30^ Similarly, the efforts of VHANCDSP to ensure RM connectivity and adherence make it well-equipped to study implementation of alert-based RM care, with in-person visits prompted by RM findings.^8^ As VHANCDSP continues to expand, it is well-positioned to generate insights that can drive evidence-based practices for managing patients with CIEDs.

## Limitations

The VHANCDSP’s current infrastructure should be considered in the context of current limitations and planned strategies to overcome some of them. First, although VHANCDSP knows if a patient has been seen at an in-person clinic visit, it does not currently have data from interrogations or programming performed during those visits within its data system. However, RM transmission reviews include all data since the patient’s last remote transmission and these data are available in the VHA’s national electronic health record. Further, VHANDSP is adding software in 2025 to enable clinician visits to be documented in its Web-based application, including both discrete CIED data and PDFs from programmers, along with local clinicians’ interpretation of those data. Second, although VHANCDSP has authority to ensure optimal patient care at local VHA clinics, VHANCDSP to date has not monitored the quality of patient care beyond the clinic responses to recalls and alerts. With the implementation of software for in-clinic visits, future initiatives are planned to drive evidence-based care such as adherence to recommendations for ICD programming^31^^,^^32^ and reducing right ventricular pacing, given its association with adverse clinical outcomes.^33^^,^^34^

## Conclusion

RM is a critical component of providing high-quality care to patients with CIEDs to achieve optimal cardiovascular outcomes. Managing the immense workload of an individual RM clinic can be supported by a centralized RM infrastructure; although there are limited peer-reviewed publication data about centralized RM, 1 study of a centralized RM system in France found an association with reduced mortality.^35^ In addition to serving as a central data source, the VHANCDSP reviews RM transmissions, promotes RM participation and adherence, and provides guidance on management of recalls to reduce administrative workload for frontline clinicians, enabling the provision of high-quality, evidence-based CIED care. By continuously improving workflows and integrating evidence-based practices, the VHANCDSP not only enhances care delivery but also generates valuable data that can inform future clinical strategies. The ability of the program to scale and adapt to emerging challenges, such as device recalls or increases in patient volume, positions it as a leader in optimizing RM care for veterans with CIEDs.

## Disclosures

The authors have no conflicts of interest to disclose.
